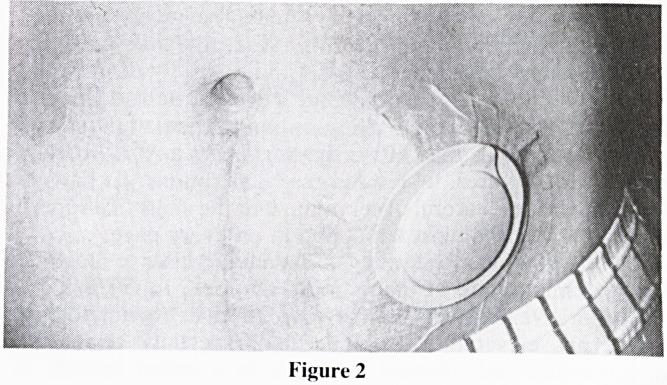# The Conseal Continent Colostomy Appliance

**Published:** 1989-05

**Authors:** Gill Down, David Leaper

**Affiliations:** Stoma Sister; Senior Lecturer, Department of Surgery, Southmead Hospital, Bristol BS10 5NB


### The Conseal Continent Colostomy Appliance: Southmead Hospital Experience

**Gill Down** Stoma Sister
**David Leaper** Senior Lecturer, Department of Surgery, Southmead Hospital, Bristol BS10 5NB

### BACKGROUND

The concept of a continent stoma is an attractive one. This has been achieved with varying success for ileostomies made with a Koch pouch but not for colostomies. Permanent ileostomies, and to a proportionally lesser extent colostomies also, are becoming increasingly rare following increased expertise in sphincter saving operations, mucosal proctectomy and the success of the J- and W-ileoanal pouches. The number of permanent colostomies, particularly among older patients, is still substantial in each general surgeon's practice. To them should be added the patients undergoing an emergency Hartmann's procedure who may wait several months before colostomy closure. This latter operation is often difficult and the decision to proceed may be avoided by patient, surgeon or both.

In the United Kingdom patients with an end, left-iliac fossa colostomy are usually left to be incontinent into a bag-appliance which they wear permanently and requires patience and long-term care from a stomatherapist. By contrast in the United States of America patients are taught the technique of colostomy irrigation to avoid the use of expensive permanent bulky bag-appliances which they have to pay for personally but this can be a messy, time-consuming procedure taking up to an hour a day. Only the younger, motivated and capable patients can manage irrigation. In both groups many patients can predict long periods (longest in those who irrigate) when their colostomy will not act apart from passage of flatus and it is for these patients that a continent device would be welcome so that they need not wear a bulky appliance between colostomy faecal actions.

Several operative techniques for this have been devised using inflatable plugs and artificial "sphincters". (I can remember 2 or 3 occasions of implanting a magnetic ring under the skin around a colostomy some years ago. This allowed colostomy continence with a metallic ring placed on the skin over the subcutaneous magnet. Flatus could easily be released by raising the metal ring. The technique was attractive but was eventually abandoned but my memory of it was the disconcerting way the magnet seemed to pull the needle and needle holder out of one's hand when suturing it in place!—DJL).

### THE NEW CONSEAL APPLIANCE

In this paper we report our experience of a new device; the Conseal continent colostomy appliance made by Coloplast Ltd. The device is a disposable system consisting of a conventional 10 cm diameter carboxymethylcellulose base plate covered by a microporous adhesive and the novel colostomy plug. The base plate can be cut to the size of the stoma and around this hole is an external coupling flange which allows an effective seal to a colostomy bag or the colostomy plug. The plug is made of an open-cell polyurethane foam which allows gas to pass and be released silently and without smell because of an inbuilt carbon filter. Because of its bell shape, 3.5–4.5 cm length and 2.4 cm distal diameter, it prevents passage of fluid and faeces. The plug is fixed to a water-impermeable cover and compressed in a water-soluble film which distintegrates within a few seconds after insertion to expand to its full size (figure 1). A new plug may be applied after irrigation or a bowel action without removing the base plate (figure 2).

A preliminary communication from Denmark (Burcharth *et al*, 1986) reported that in 53 patients faecal continence and passage of flatus without noise or odour was achieved in 90%. The median application period until the plug became obstructed by mucus or faeces was 8 hours (range 5–24 hours). It was more successful in patients who irrigated their colostomies whereas non-irrigators had to replace the plug with a bag at night.

A larger study was started in the United Kingdom in 1986 involving 12 centres and recruiting patients with an end left-iliac fossa colostomy who were well practiced in their stoma care. Southmead Hospital was one of these centres and we report our experience involving nine patients.

### PATIENTS AND TRIAL METHODS

After gaining local ethical committee approval patients being managed by the Southmead stoma sister (GD) were invited to participate after gaining their informed consent. Patients were asked to complete a questionnaire about their colostomy management seven days before the 4 week trial assessment period and seven days after. This allowed for comparisons to be made during the 4 week period when the plug was used. Only patients over 18 were admitted and were able to withdraw at any time from the study for any reason. Those with colostomy stenosis, a colostomy diameter of greater than 3.5 cm, protrusion of more than 2.5 cm or known residual disease were excluded.

Data was collected onto proformata to investigate skin and stoma problems and compliance with the device. An assess-

Figure 1

Figure 2

**Table 1**

**Demographic data ($n = 9$)**

| | | | |
|---|---|---|---|
| Men | 5 | Women | 4 |
| Age range | 44–67 | | 59–70 |
| *Reason for colostomy* | | | |
| Cancer | 8 | Other | 1 |
| *Stoma level (cms)* | | | |
| Flush | 5 | Raised 0–2 cm | 4 |
| Raised 0–2 cm | | | |
| *Stoma Diameter (cms)* | | | |
| Up to 2.0(4) | 2.1–2.5(3) | 2.6–3.0(1) | 3.1–3.5(1) |

**Table 2**

**Patient evaluation of device ($n = 9$)**

| | *Yes* | *No* | *Unsure* |
|---|---|---|---|
| Useful | 6 | 3 | — |
| Increased quality of life | 5 | 4 | — |
| Prefer conseal to conventional stoma bag system | 5 | 4 | — |
| Would use conseal regularly | 4 | 3 | 2 |

ment form, completed by the patients, allowed their evaluation of the device to be recorded with particular reference to quality of life and any advantages or disadvantages.

### RESULTS

Nine patients were admitted to the trial and their demographic data is shown in Table 1. Five completed the four week evaluation period successfully and were keen to continue using it (particularly as it allowed quiet passage of flatus). But four withdrew. The reasons for withdrawal were: that the device conferred no advantage to one patient; there were two mechanical causes for failure—faecal leakage and plug extrusion; and one patient who was generally unwell and lost motivation to complete the trial. Only one patient had skin problems with the system (erythema) and withdrew because of leakage. Patients' evaluations are shown in Table 2.

Some of our patients developed a sensation of fullness when they had a plug in place and were able to predict when they needed to attach a bag prior to a colostomy faecal passage. Some of them had an accident between removing a plug and attaching a bag.

### DISCUSSION

It should be remembered that all nine patients were content with their conventional appliance and therefore evaluation was loaded against showing a clear advantage to any change of stoma management. Nevertheless the majority of patients felt the device had advantages and preferred it. All but one patient had complete continence between bowel action when the Conseal device was in place. No complications occurred except minor skin erythema in the patient with faecal leakage.

The device offers an advantage to some patients who are expert in managing their stomas independently. None of our patient irrigated their colostomies but we feel even for some of them there was an advantage to the Conseal device.

### REFERENCE

BURCHARTH, F., BALLAN, A., KYLBERG, F. *et al* (1986) The colostomy plug: a new disposable device for a continent colostomy. *Lancet* **i**, **ii**, 1062–1063.

## Figures and Tables

**Figure 1 f1:**
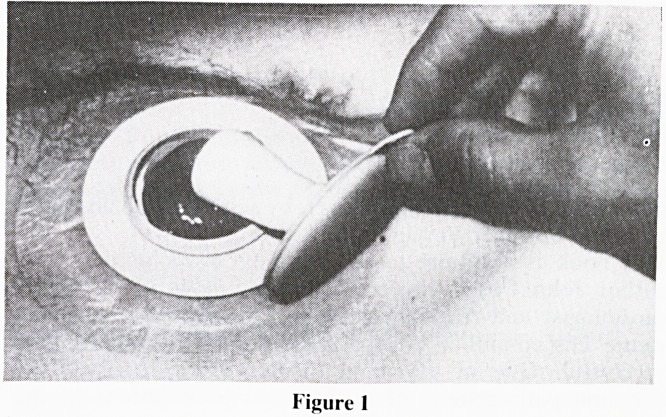


**Figure 2 f2:**